# Printing Cell Embedded Sacrificial Strategy for Microvasculature using Degradable DNA Biolubricant

**DOI:** 10.1002/anie.202417510

**Published:** 2024-11-27

**Authors:** Jiezhong Shi, Yifei Wan, Haoyang Jia, Gregor Skeldon, Dirk Jan Cornelissen, Katrina Wesencraft, Junxi Wu, Gail McConnell, Quan Chen, Dongsheng Liu, Wenmiao Shu

**Affiliations:** ^1^ Key Laboratory of Bioorganic Phosphorus Chemistry & Chemical Biology (Ministry of Education) Department of Chemistry Tsinghua University Beijing 100084 China; ^2^ Department of Biomedical Engineering University of Strathclyde Glasgow G4 0NW United Kingdom; ^3^ SINOPEC Key Laboratory of Research and Application of Medical and Hygienic Materials SINOPEC Beijing Research Institute of Chemical Industry Co., Ltd. Beijing, 100013 China; ^4^ Department of Physics, SUPA University of Strathclyde Glasgow G4 0NG United Kingdom; ^5^ State Key Laboratory of Polymer Physics and Chemistry Changchun Institute of Applied Chemistry, Chinese Academy of Sciences Changchun 130022 China

**Keywords:** Microvasculature, DNA biolubricant, 3D printing, Tissue engineering

## Abstract

Microvasculature is essential for the continued function of cells in tissue and is fundamental in the fields of tissue engineering, organ repair and drug screening. However, the fabrication of microvasculature is still challenging using existing strategies. Here, we developed a general PRINting Cell Embedded Sacrificial Strategy (PRINCESS) and successfully fabricated microvasculatures using degradable DNA biolubricant. This is the first demonstration of direct cell printing to fabricate microvasculature, which eliminates the need for a subsequent cell seeding process and the associated deficiencies. Utilizing the shear‐thinning property of DNA hydrogels as a novel sacrificial, cell‐laden biolubricant, we can print a 70 μm endothelialized microvasculature, breaking the limit of 100 μm. To our best knowledge, this is the smallest endothelialized microvasculature that has ever been bioprinted so far. In addition, the self‐healing property of DNA hydrogels allows the creation of continuous branched structures. This strategy provides a new platform for constructing complex hierarchical vascular networks and offers new opportunity towards engineering thick tissues. The extremely low volume of sacrificial biolubricant paves the way for DNA hydrogels to be used in practical tissue engineering applications. The high‐resolution bioprinting technique also exhibits great potential for printing lymphatics, retinas and neural networks in the future.

## Graphical abstract

We developed a general PRINting Cell Embedded Sacrificial Strategy (PRINCESS) and successfully fabricated microvasculatures using degradable DNA biolubricant. This is the first demonstration of direct cell printing to fabricate microvasculature. Utilizing the DNA hydrogel as biolubricant, we can print a 70 μm endothelialized microvasculature and cell‐laden tissue constructs with branched microvasculatures.

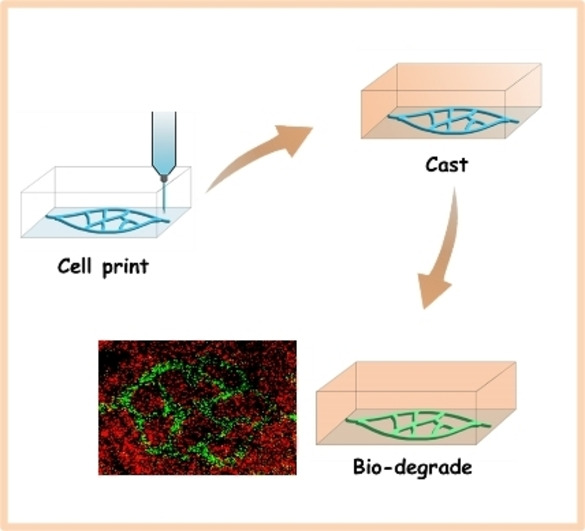

## Introduction

Microvasculature (arterioles, capillaries, and venules) allows the exchange of oxygen, carbon dioxide, nutrition, endocrine signals and waste to and from all the cells. This process is essential for the continued function of cells in the tissue. The microvasculature must permeate the entirety of tissues to allow sufficient access to this exchange for all cells in the body, lest they become dysfunctional or die. The intricate structure of the microvasculature has meant its replication in vitro has been challenging, and its development is fundamental in the fields of tissue engineering,^[1]^ organ repair^[2]^ and drug screening.^[3]^ An established strategy to create microvasculature in vitro is using microfluidic devices based on standard microfabrication techniques such as thin film deposition, lithography and etching.[^4^, ^5^] Utilizing microfluidics, one approach is to seed endothelial cells inside the microchannels to form microvasculature,^[6]^ but the fabrication of 3D microvasculature requires layer‐layer assembly,[^7^, ^8^] which is time‐consuming and can result in delamination or other defects. Another approach is to emulate the microphysiological environment using microfluidic devices by perfusing culture medium in two parallel microchannels, and promoting the assembly of endothelial cells encapsulated in the hydrogel matrix between the microchannels into 3D microvasculature.[^9^, ^10^, ^11^] However, the structure of the resulting microvasculature is uncontrollable and the hydrogels are limited to naturally occurring hydrogels, such as fibrin and collagen.

Alternatively, another elegant approach is based on a sacrificial strategy to fabricate microvasculature in 3D matrix.[^12^, ^13^, ^14^, ^15^, ^16^, ^17^, ^18^, ^19^, ^20^] Rigid, sacrificial filaments are printed first and cast into a hydrogel matrix, before the sacrificial filaments are removed leaving rudimentary microvascular channels. Numerous sacrificial materials such as gelatin,^[13]^ sugar,^[14]^ shellac,^[15]^ PNIPAM^[16]^ and Pluronic F127^[18]^ have been developed to generate microvasculature. By utilizing 3D printing technology, microvasculature with various designs has been fabricated.[^17^, ^18^, ^19^, ^20^, ^21^, ^22^] However, all previous sacrificial techniques entailed first constructing the hollow channels, with subsequent cell seeding. This two‐step process often involves delicate experiment steps including sacrificial gel removal and subsequent cell seeding by perfusion, and hence cannot achieve spatial and density control of the seeded cells. Moreover, the uncontrollable cell perfusion in the microchannels affects the cell adhesion and the integrity of the resulting microvasculature, resulting in low reproducibility. At present, fabrication of endothelialized microvasculature with a diameter smaller than 100 μm is still a significant challenge.^[23]^

**Scheme 1:**
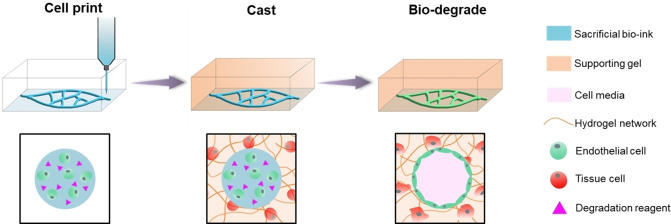
Illustration of PRINting Cell Embedded Sacrificial Strategy (PRINCESS). A sacrificial biolubricant which is embedded with endothelial cells and degradation reagents is bioprinted into the desired microvasculature structure at high resolution. A supporting hydrogel is then cast over the sacrificial structure, which itself can contain cells. Degradation occurs within the sacrificial filaments under controlled conditions, leaving an appropriately endothelialized lumen.

Here, we developed a new PRINting Cell Embedded Sacrificial Strategy (PRINCESS) to fabricate microvasculature in tissue constructs. In contrast to the sacrificial strategy whereby hollow channels are created before cells are seeded, PRINCESS directly prints viable cells to produce a microvascular network. As shown in Scheme 1, the endothelial cells encapsulated in the sacrificial biolubricant with degradation reagents are first printed, before being cast over by tissue cells encapsulated in another supporting gel. After degradation of the sacrificial biolubricant with the encapsulated degradation reagents, the cells remain within the consequent channel, and a tissue construct with endothelialized microvasculature is formed. In this strategy, it is important to ensure the degradation process is not harmful to encapsulated cells. Therefore, the control of the rapid bio‐degradation of the sacrificial biolubricant is crucial.

To this end, DNA hydrogel is chosen as the sacrificial biolubricant in our PRINCESS. DNA hydrogel[^24^, ^25^, ^26^, ^27^, ^28^, ^29^, ^30^, ^31^, ^32^] is an ideal bio‐degradable material which can be degraded by a range of enzymes under physiological conditions. DNA hydrogel is also known to exhibit excellent potential properties for bioprinting of microvasculatures.^[33]^ Due to its strong supramolecular interactions and kinetically interlocking gelation mechanism, the DNA hydrogels possess excellent shear‐thinning properties, which enable them to be dispensed through a tiny printing nozzle easily. Also, the lubrication properties of DNA hydrogel can ensure the high viability of printed cells, providing the possibility to achieve high‐resolution bioprinting. Finally, supramolecular hydrogels based on DNA also possesses self‐healing properties which are found to be suitable for printing complex, branched vascular microstructures.^[34]^ Therefore, the aim of this work was to construct microvasculatures through PRINCESS using degradable DNA biolubricant.

## Results and Discussion

### 1 System Design

**Scheme 2:**
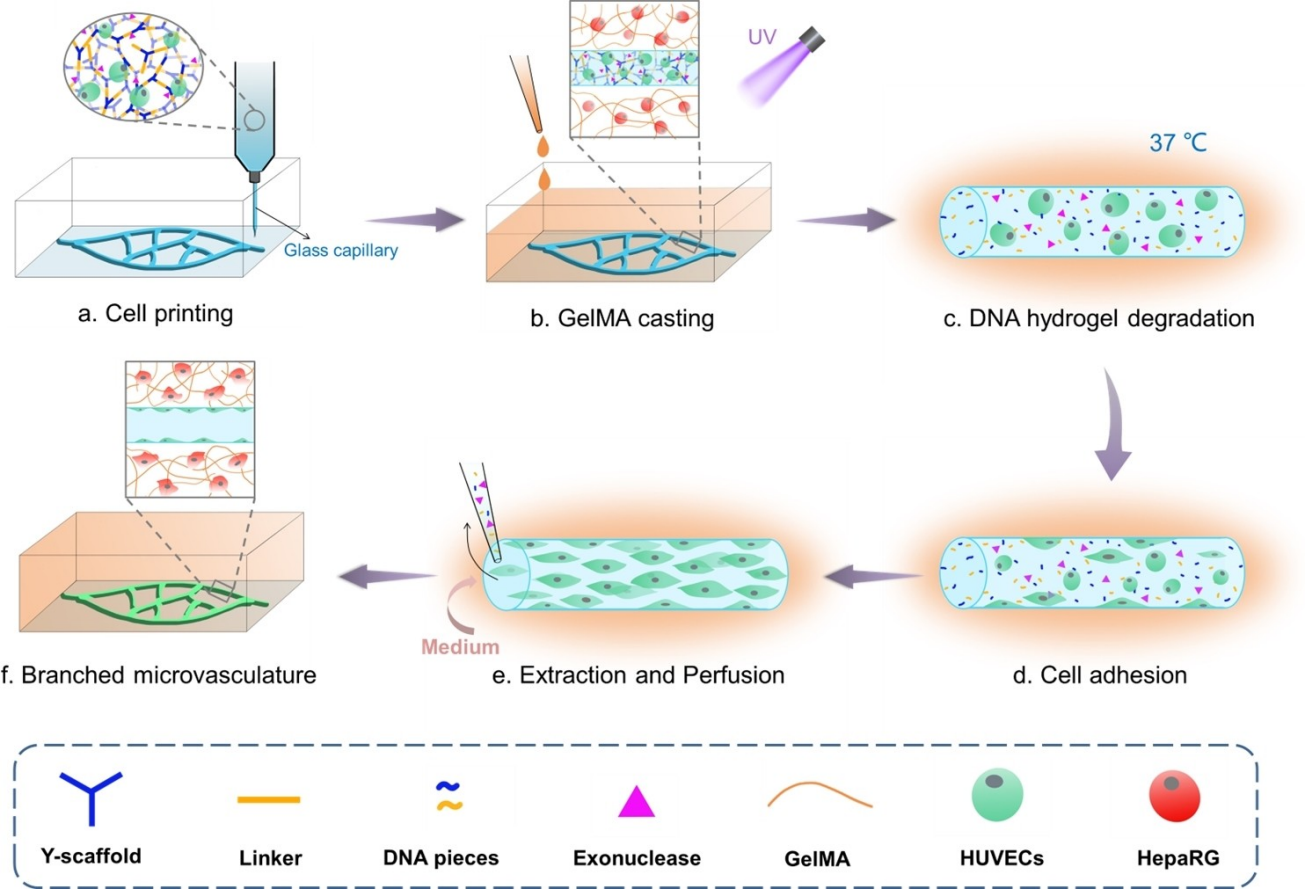
Fabrication of branched microvasculature using DNA hydrogel as sacrificial biolubricant in PRINCESS.

To demonstrate the capability of the PRINCESS for microvasculature fabrication, we designed the following experimental protocol as shown in Scheme 2. DNA hydrogel with encapsulated human umbilical vein endothelial cells (HUVECs) and exonuclease served as the sacrificial biolubricant and was printed using a glass capillary to form thin filaments. Gelatin methacrylate (GelMA) with hepatoma derived cell line HepaRG was then cast on the printed microstructure and crosslinked under UV light. GelMA is used as supporting gel due to its favorable biological properties and its proclivity for cell attachment.^[35]^ Next, the DNA hydrogel was then gradually degraded enzymatically in a cell incubator (37 °C and 5 % CO_2_), allowing the embedded HUVECs to slowly migrate and adhere to the printed lumen surface of GelMA. Finally, after extracting the degraded sacrificial biolubricant with non‐adherent HUVECs, DNA pieces and exonuclease, the microvasculature is perfused with cell culture medium to promote cell growth and the microvascularized liver construct is eventually formed.

### 2 Advantages of DNA Hydrogel as Sacrificial Biolubricant

**Figure 1:**
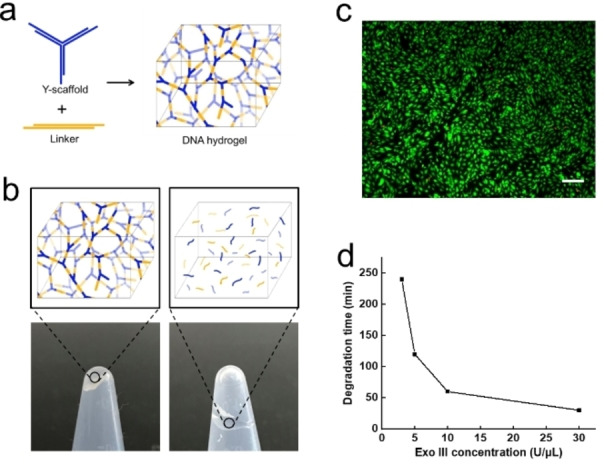
Gelation and degradation of DNA hydrogel. a. Gelation of DNA hydrogel by mixing two building blocks (Y‐scaffold and Linker); b. Enzymatic degradation of DNA hydrogel by Exo III; c. Live/dead staining image of HUVECs in 24‐well plate with 150 U Exo III after 3d culture. Scale bar 200 μm; d. Degradation time of DNA hydrogel with different Exo III concentration.

The degradability of DNA hydrogel was investigated as controlling this process would be essential in the creation of the microvascular structures and maintaining cell viability. The DNA hydrogel is formed by mixing two complementary construction parts.^[25]^ As shown in Figure 1a, one part is Y‐scaffold formed of three single DNA strands (each containing 40 nucleotides), and the other part is a linear duplex linker with 44 nucleotides in length (Sequences of ssDNA are shown in Table S1). The “sticky ends” of the Y‐scaffold and the linker of the DNA parts are complementary, so that they will lead to hydrogel gelation quickly when they are mixed. To degrade DNA hydrogel, the degradation reagent Exo III with the specific activity of 150,000 units/mg was introduced to the sacrificial ink. Exo III is an exonuclease enzyme which can degrade the mononucleotides from 3'‐hydroxyl termini of duplex DNA,^[36]^ resulting in the transition from hydrogel to solution. The change of DNA hydrogel network during degradation is illustrated in Figure 1b. This degradation process occurs in biologically amenable conditions and Exo III is demonstrated to be nontoxic to HUVECs (Figure 1c) by live/dead staining using Fluorescein Diacetate (green, live) and Propidium Iodide (red, dead), indicating the DNA hydrogel has great degradability. The degradation time of DNA hydrogel is also explored with different concentrations of Exo III. As shown in Figure 1d, degradation time decreases with the increase of Exo III concentration, and DNA hydrogel can be digested within 30 min when 30 U/μL Exo III is introduced. This result shows that the degradability process is biocompatible, rapid and controllable. It is notable that endonucleases are not suitable for this system because the DNA hydrogel cannot be degraded over a day using endonucleases.

**Figure 2:**
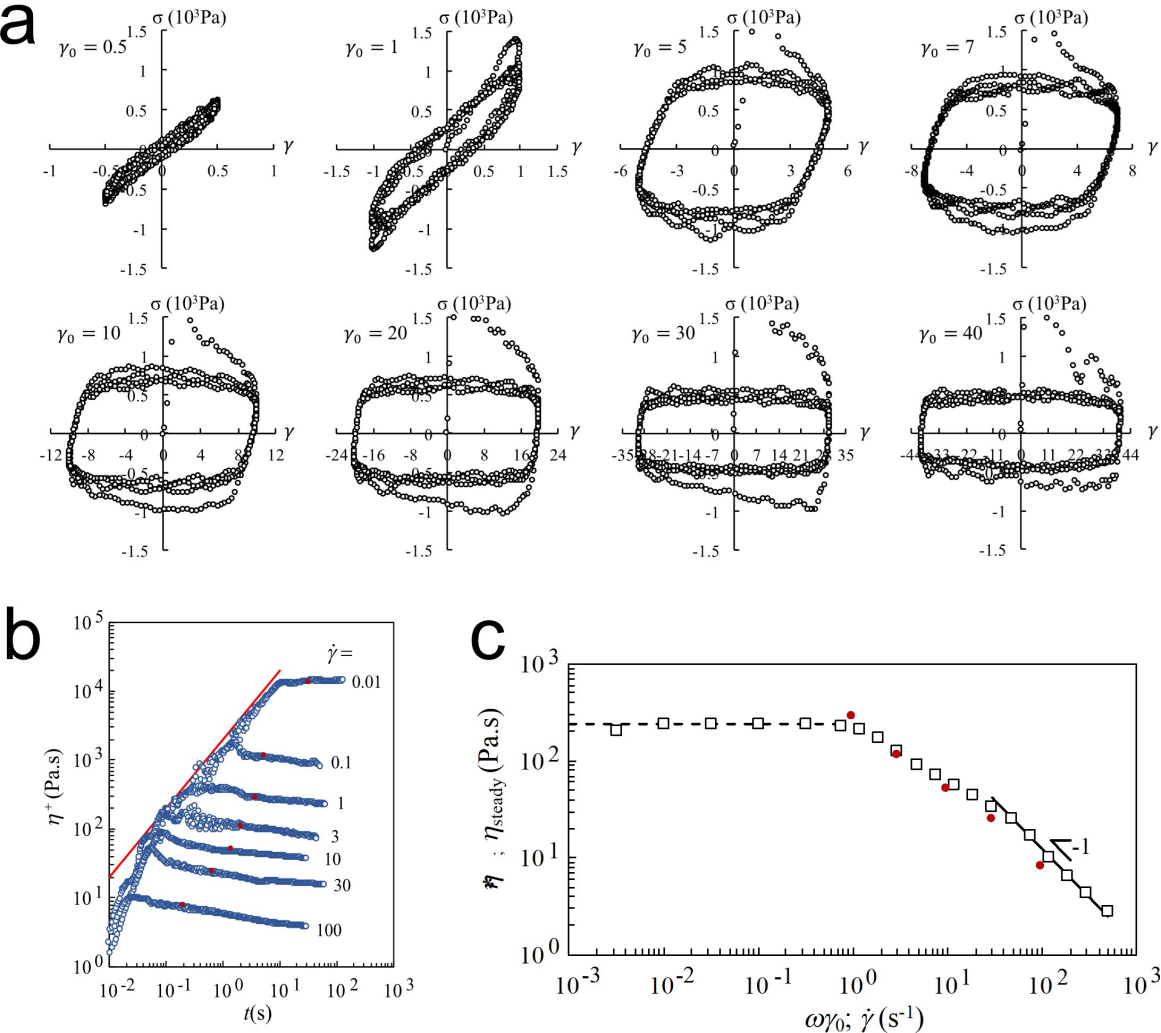
Lubrication property of DNA hydrogel; a. Stress‐strain Lissajous curves: at a strain $\gamma_{0}$ of 0.5, the DNA hydrogel is still elastically dominant, the ellipse in the curve is more oblate; as the strain increases to 5, the DNA hydrogel shows plastic behaviour and the curve approaches a diamond shape; the maximum value of the stress is 500–1000 Pa; the strain continues to increase and the stress remains at 500–1000 Pa; continue to increase the strain to 40 and the curve becomes rectangular. The maximum stress decreases and stabilises to approximately 500 Pa; b. In the initiation shear test, at different shear rates$\;\dot{\gamma \;}$ , the transient shear viscosity $\eta^{+}$ of the sample increased with strain for a short time (shown by the red line); c. Contrast plots of complex viscosity 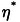 relative shear rate $\omega \gamma_{0}$ (hollow squares) at fixed ω =10 rad/s with plots of steady shear viscosity η_steady_ (as indicated in red sphere symbols in panel b) relative shear rate $\dot{\gamma }$ (solid circles); at a shear rate $\dot{\gamma }$ ≥10 s^−1^, $\eta =\sigma_{steady}/\dot{\gamma }$ , $\sigma_{steady}≅1000\;\;Pa$ .

We also explored the lubrication property of DNA hydrogel because it can protect cells during printing process. In the rheological test, the non‐linear relationship performed an unstable flow in the sample, which may be owing to shear banding in the sample itself or a slip between the sample and the plate wall. The non‐linear change of stress with strain was classified in the stress‐strain curve of the sample (also called the Lissajous curve, Figure 2a). In the linear region, the stress‐strain curve is usually an ellipse. With increasing the strain to 40, and the stress‐strain curve gradually changes from a rhombus to a rectangle. The maximum value of stress gradually decreases from 1000 Pa to around 500 Pa. In other words, increasing the strain and strain rate did not increase the stress, but reduces the stress. When the peak strain is between 5 and 10, the plot shows varying stress fluctuations. Even when the structure is finally stable, there is still a large‐amplitude continuous stress fluctuation phenomenon. This phenomenon may be caused by the contact and slippage between the sample and the plate during the oscillation process when the appropriate shear rate range is changed.^[37]^ The randomness of the change caused the irregularity of stress fluctuations. In steady rheology test, when reaching a steady‐state, the viscosity changes with the shear rate. As shown in Figure 2b, in a short period of starting, the transient shear viscosity of the material increases with strain. Once the system enters a steady‐state flow, the viscosity reaches a fixed value. However, when the shear rate is above 3, the viscosity of the system keeps decreasing with the increase of time, which proves the non‐steady flow owing to either the shear banding or the wall slip. As a result, a stress force σ_steady_ between 500 Pa and 1000 Pa is generated, and when this force is a constant value:

$$\eta =\sigma_{steady}/\dot{\gamma }$$

In the dynamic rheological test of DNA hydrogel (Figure 2c, hollow squares), we observe a region (as indicated in line):

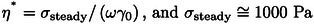




In the steady rheological test of DNA hydrogel (Figure 2c, solid circles), we also observe a region:

$$\eta =\sigma_{steady}/\dot{\gamma },\;\mathrm{and}\;\sigma_{steady}≅1000\;\;Pa$$

Thus, both dynamic and steady rheological tests show that the DNA hydrogel slips or delaminates with the plate at a stress of ~1k Pa, resulting in the reduction of internal shear rate and lubrication property of the DNA hydrogel. In theory, all of the supramolecular DNA hydrogels can be a biolubricant due to their excellent shear‐thinning property, but the ability to be a biolubricant also depends on the DNA sequence, length and assembly buffer, etc.

### 3 3D Printing Various Patterns Using DNA Biolubricant

**Figure 3:**
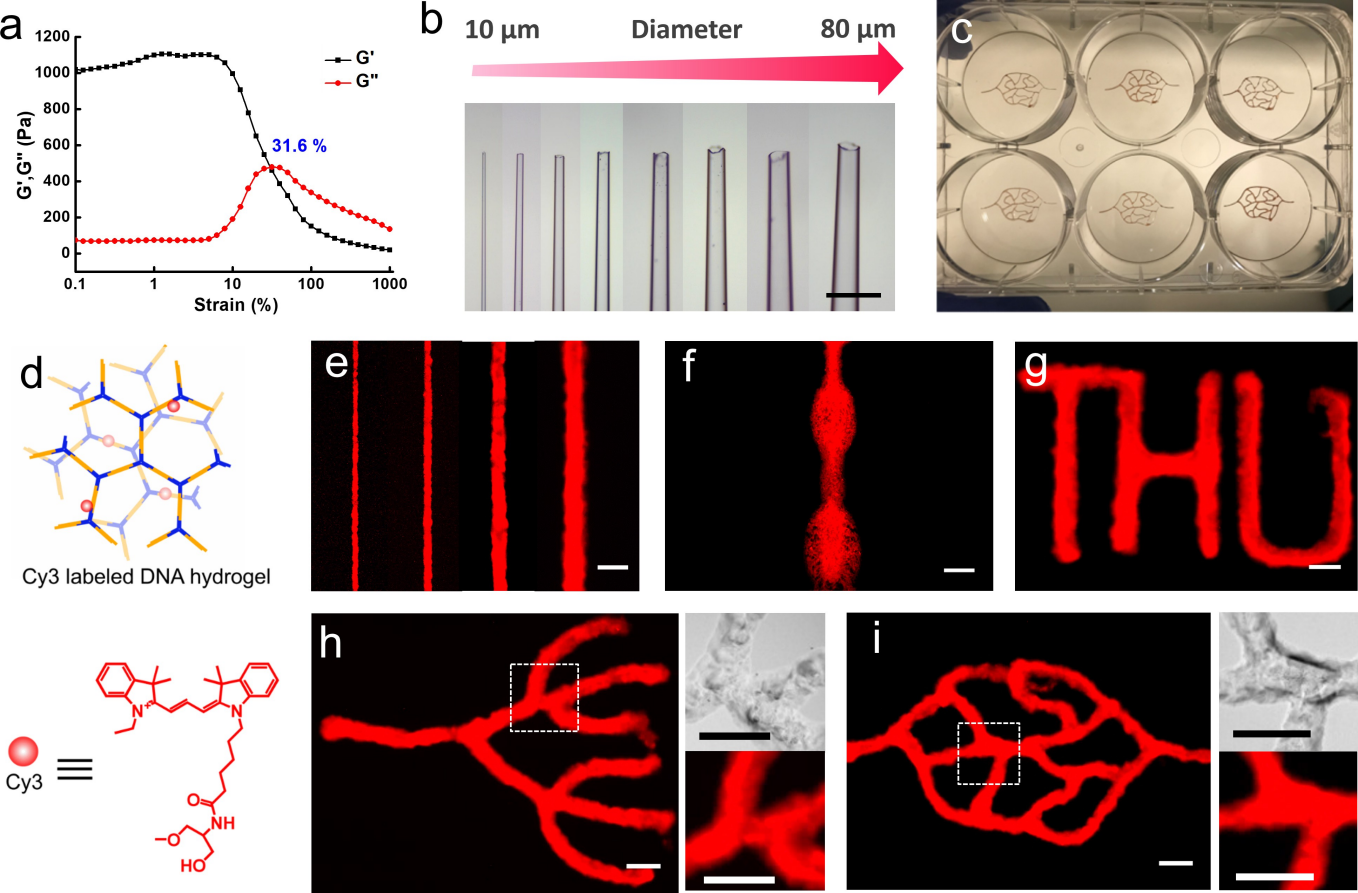
Printability of the DNA hydrogel. a. Rheological characterization of 3.8 wt % DNA hydrogel in 1×PBS buffer with different strain at a fixed ω =2π rad/s; b. Glass capillaries with different diameters (10, 20, 30, 40, 50, 60, 70, 80 μm); c. Printed vascular structure in a six‐well plate; d. Scheme of Cy3 labeled DNA hydrogel; e–i. Widefield fluorescent images of printed Cy3 labeled DNA hydrogel; e. Filaments with different diameters (25, 50, 100, 150 μm); f. a continuous filament with different diameters; g. Branched letters “THU”; h, i. Branched vascular structure (Right pictures are optical and fluorescent images in the area of dotted line). Scale bar 200 μm.

We then explored the printability of the DNA hydrogel. The mechanical strength of DNA hydrogel was first characterized with the strain sweep measurements at fixed *ω*=2π rad/s using a shear rheometer. Figure 3a shows that the shear‐storage modulus *G*′ is significantly higher than the shear‐loss modulus *G*“ when the strain is below 10 %, indicating that the formation of the hydrogel is verified as designed. When the strain increases, *G*′ decreases and *G*” increases dramatically, and *G*“ becomes higher than *G*′ as the strain exceeds 31.6 %, showing the hydrogel has great shear‐thinning property and can be used for high‐resolution printing.^[38]^ Due to this shear‐thinning property, different structures of DNA hydrogel were printed using a custom‐built extrusion based 3D bioprinter. This bioprinter represents an adapted, extrusion‐based version of a previously developed microvalve‐based bioprinter used to bioprint human cells including human induced pluripotent stem cells.^[39]^ In order to print thin filaments, a glass capillary is connected to the printer and used as nozzle. As shown in Figure 3b, designed diameters (from 10 μm to 80 μm) can be obtained by pulling the glass tube using a P‐1000 Micropipette puller (Sutter Instrument) and cutting the glass capillary under an optical microscope. Using the printer, vascular structures were printed in a six‐well plate (Figure 3c). For visualization, 6 % Cy3 fluorescent dye was introduced into the DNA hydrogel. As shown in Figure 3d, Cy3 is covalently modified on the duplex linker. (MALDI‐TOF characterization is shown in Figure S4). The size of printed filaments can be controlled by adjusting the diameter of glass capillary, and the thinnest filament printed was approximately 25 μm using a 20 μm glass capillary (Figure 3e), which was attributed to the great shear‐thinning property^[40]^ of DNA hydrogels. The slight expansion of the filament size may be because the DNA hydrogel undergoes a short period of sol‐to‐gel transition after printing through the glass capillary due to its shear‐thinning property. We can also print a continuous filament with different diameters by altering the printing speed during printing process. As shown in Figure 3f, a filament with alternate diameter is printed. Branched microstructures, including simple letters “THU” and complicated vascular structures are printed using 40–80 μm glass capillaries (Figure 3g–i). The sizes of these microstructures are all around 2 mm ×2 mm, which could not be fabricated manually or using standard 3D printing extrusion nozzles. Optical and fluorescent images of branched parts of Figure 3h and 3i are also displayed. These images highlight that disparate filaments merged together and left no gap between them, demonstrating that utilizing the great self‐healing property of the DNA hydrogel allows the creation of continuous branched structures.

### 4 Formation of Microvascular Channels and Bioprintability of DNA Biolubricant

**Figure 4:**
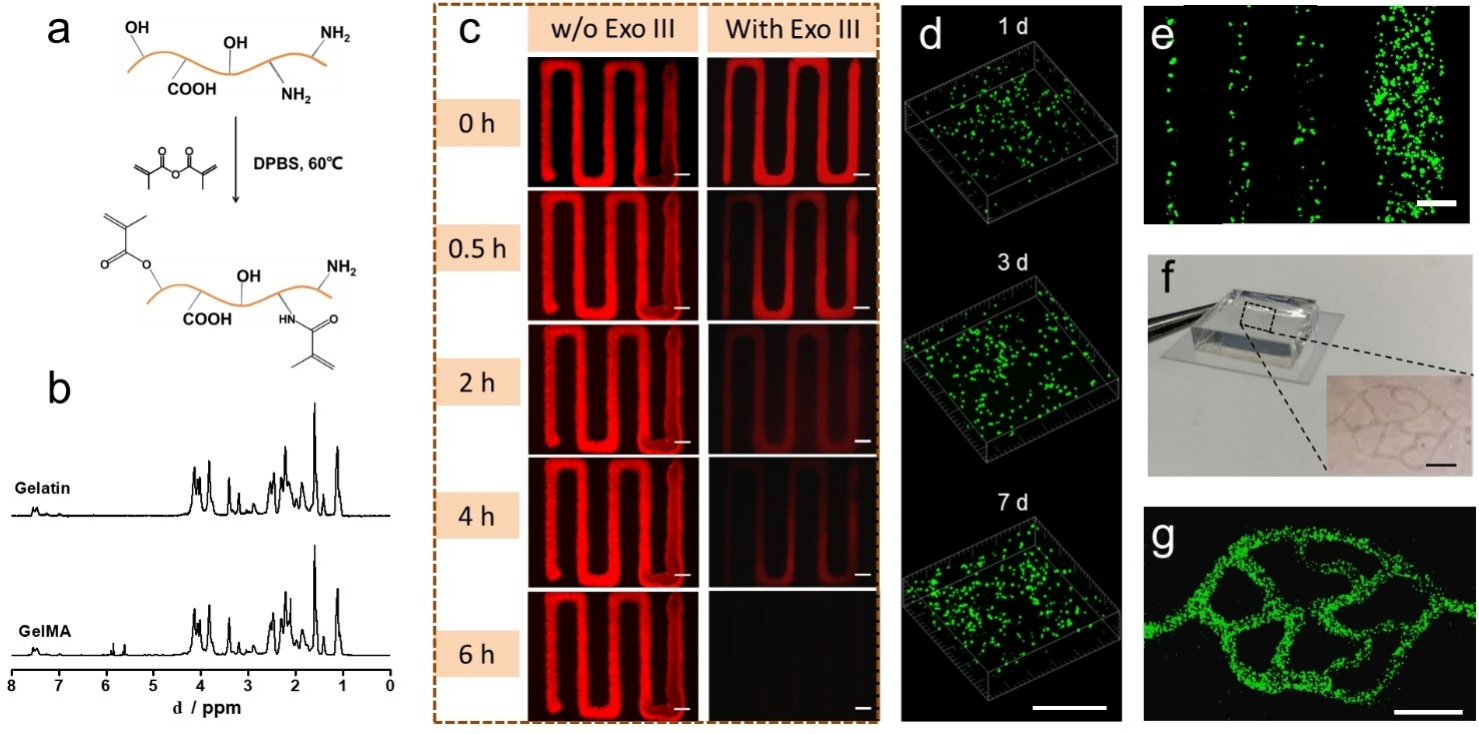
GelMA cast on the printed DNA hydrogels. a. Reaction of gelatin and methacrylic anhydride for synthesizing GelMA; b. ^1^H NMR spectra of gelatin and GelMA; c. Fluorescent images of printed Cy3 labeled DNA hydrogel cast by 15 wt % GelMA with or without 5 U/μL Exo III in the incubator after 0 h, 0.5 h, 2 h, 4 h and 6 h. Scale bar 200 μm; d. Live/dead staining images of HUVECs printed in the DNA hydrogel after 1 d, 3 d and 7 d. Scale bar 200 μm; e. Confocal epifluorescence images of HUVECs with different diameters (50, 100, 200, 400 μm). Scale bar 200 μm; f. Pictures of printed tissue construct. Zoom‐in picture is printed branched vascular structure using 3.8 wt % DNA hydrogel (with 2×10^7^/mL HUVECs) cast by 15 wt % GelMA. Scale bar 1 mm; g. Confocal epifluorescence images of HUVECs within branched vascular structure. Scale bar 1 mm.

Next, we investigated the formation of microvascular channels in a supporting gel of GelMA after degradation of printed DNA structures. GelMA is synthesized by the reaction of gelatin and methacrylic anhydride.^[41]^ As shown in Figure 4a, methacryloyl substitution groups were grafted onto the reactive amine and hydroxyl groups. This reaction is characterized by ^1^H NMR . As illustrated in Figure 4b, compared with gelatin, there are three new peaks for GelMA at δ=2.10 ppm, δ=5.62 ppm and δ=5.87 ppm, which is assigned to introduced methacrylate.^[42]^ This result indicates that methacrylate has been successfully grafted to the gelatin molecules, and the degree of methacrylation was calculated to be 57.4 %.[^43^, ^44^]

Subsequently, zig‐zag structures were printed using the pure DNA hydrogel and cast with 15 wt % GelMA. As shown in Figure 4c, the microvascular structure keeps its original shape and does not collapse after 6 hours, and even maintained its structure for 7 days (Figure S5), showing that the DNA hydrogel is very stable in GelMA. If the degradation reagent Exo III is premixed with DNA hydrogel before printing at room temperature, the DNA hydrogel can still retain its structure for a long period as Exo III will not be active and so the DNA will not be degraded. However, when zig‐zag structures were printed using Exo III premixed DNA hydrogel at room temperature and cast with GelMA, and then put into the cell incubator, degradation of the sacrificial microchannels was initiated. The printed structure gradually began to degrade after 30 minutes and eventually completely disappeared after 6 hours (Figure 4c), leaving microvascular channels in GelMA. These results also demonstrate that the degradability of DNA hydrogel can be controlled by the temperature.

To prove the bioprintability of our strategy, human umbilical vein endothelial cells (HUVECs) were encapsulated in the DNA hydrogel and used to print microvascular structures. At first, we characterized the viability of HUVECs after printing through the glass capillary. Figure 4d shows the 3D stack of printed HUVECs in the DNA hydrogel stained with live/dead dye after 1, 3 and 7 days. More than 99 % cells are alive in the printed hydrogel and the cell number increases significantly from day 1 to day 7, indicating that the printing process causes negligible damage to the cells which can survive and proliferate after 7 days. This is because the lubrication property of DNA hydrogel was performed under high shear stress. In the rheological test, when the hydrogel was fabricated in the capillary needle around 10–100 μm, where the shear stress reaches over 1000 Pa (calculation in Supporting Information), the wall slip effect occurs during extruding in high shear stress, which lubricates the movement of the filament and protects the cells.^[45]^

To visualize the fate of the HUVECs in the sacrificial biolubricant after bioprinting, CellTracker™ Green CMFDA Dye was used to stain HUVECs before printing. Then, printed DNA hydrogel with 2×10^7^/mL embedded HUVECs was printed into the microvascular structure and cast with 15 wt % GelMA. Figure 4e shows the HUVECs filaments printed had controllable diameters, from 50 μm to 400 μm. It is notable that the cells were lined in a near single‐cell manner in the 50 μm filament. Branched vascular structures are also printed using HUVECs (Figure 4f, g), and the confocal epifluorescence images (Figure 4g) show that the cells aligned along the printed structure homogeneously. These results indicate our strategy is readily applicable to bioprinting.

### 5 Fabrication of Cell‐laden Tissue Constructs with Branched Microvasculature

**Figure 5:**
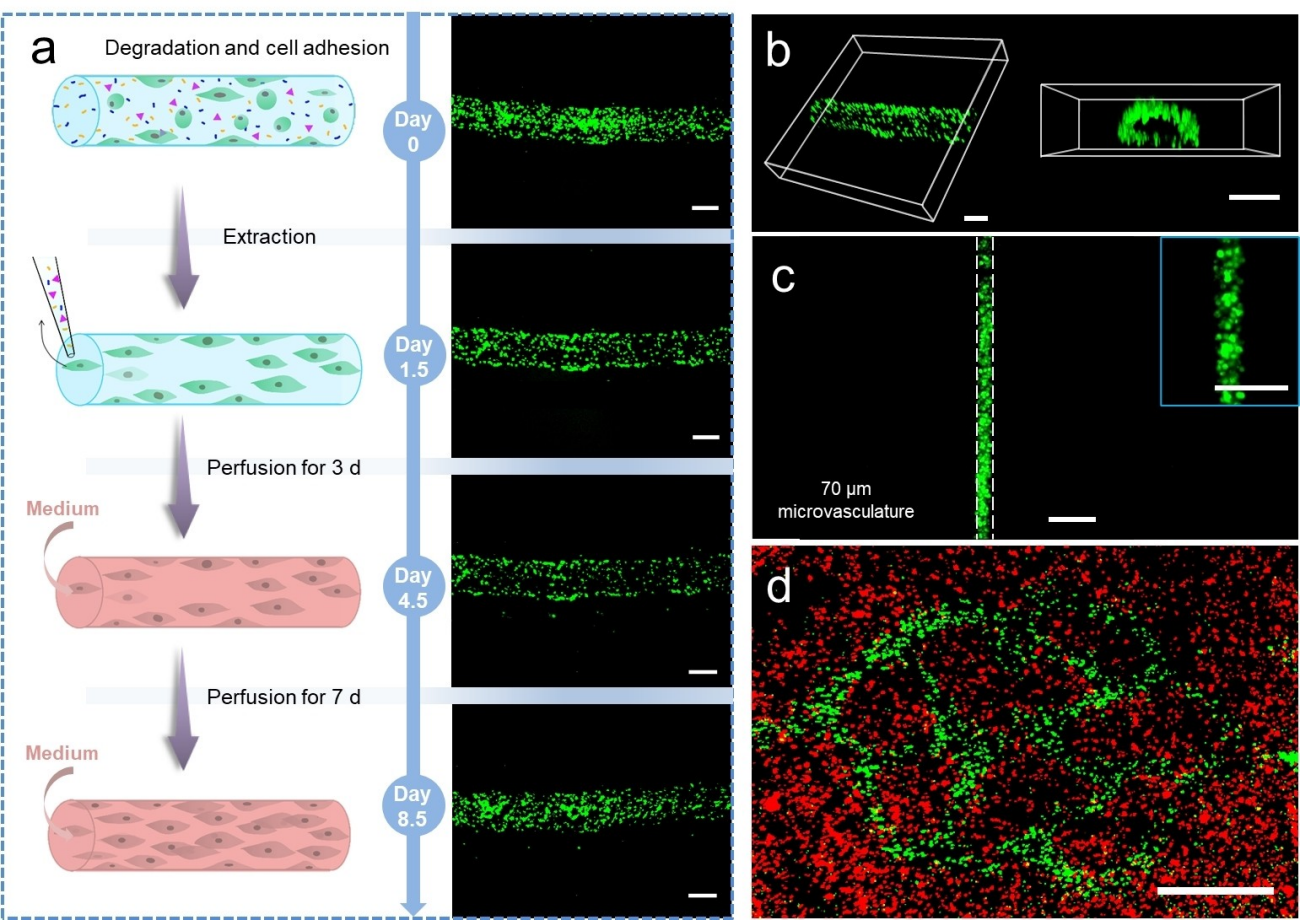
Preparation of branched microvasculature. a. Scheme and fluorescent images of extraction and perfusion process. Scale bar 200 μm; b. 3D confocal epifluorescence images of HUVECs cells lining the microchannel walls. Scale bar 200 μm; c. Fluorescent image of HUVECs in 70 μm microvasculature. Inserted picture is a zoom‐in image of microvasculature. Scale bar 200 μm; d. Confocal epifluorescence images of liver tissue construct with branched microvasculature. Red: CellTracker Red CMTPX dye labelled HepaRG cells. Green: CellTracker Green CMFDA dye labelled HUVEC cells. Scale bar 1 mm.

Finally, to form an endothelialized microvasculature, Exo III and HUVECs were embedded in the DNA hydrogel simultaneously before printing. After casting with GelMA, the construct was incubated overnight to allow DNA degradation and cell adhesion to the supporting gel. The structure was then turned upside down and put in the incubator again for 24 hours to ensure cells adhered both to the top and the bottom of the microvascular channel. Finally, non‐adherent cells, DNA pieces and Exo III were removed by aspirating the sacrificial structure. As shown in Figure 5a, the number of HUVECs decreases after removing the non‐adherent cells. The HUVECs numbers before and after extraction are counted using Image J. They are 725 before extraction and 497 after extraction, with the retention rate of 68.6 % calculated as followed:

$$Retention\;Rate=\left(\frac{HUVECs\;number\;before\;extraction}{HUVECs\;number\;after\;extraction}\;\right)\times 100\;\%=\frac{497}{725}\times 100\;\%=68.6\;\%$$

This result indicates that most cells had already attached to the channel. Moreover, the microvasculature can be further perfused with cell culture medium to promote cell growth using a custom‐built perfusion equipment and system (Figure S7). Figure 5a shows the fluorescent images of adherent HUVECs in the microvasculature after perfusing 3 and 7 days. Notably, the cell number increases considerably after 7 days, demonstrating that the perfused medium can provide nutrition and promote the proliferation of cells. As microvasculature is capable of dynamic remodeling in response to the local metabolic demand, we believe the printed vasculature can serve as a foundational vascular bed to vascularize the surrounding cell‐laden GelMA hydrogel via angiogenesis, therefore enabling complex organ morphogenesis. The benefits of the pre‐designed and perfused microvascular network are 1) providing immediate nutrient supply to the bioengineered 3D construct to ensure early‐phase cell survival, and 2) enabling organ‐specific vascular patterning to facilitate targeted organ morphogenesis. Meanwhile, this will allow fast fabrication of scalable vascular network in a larger volume at centimeter scale, which is a key step towards functional organ regeneration.

Figure 5b shows the 3D fluorescent images of HUVECs in the microvascular channel. As can be observed, HUVECs adhered to the channel walls nearly confluently and formed a hollow lumen structure, which confirms the endothelialization of the microvasculature. Moreover, a 70 μm microvasculature with numerous HUVECs attached has been fabricated using this strategy (Figure 5c). Endothelialized microvasculature with such high resolution (100 μm) has rarely been reported in previous research.

To fabricate cell‐laden tissue construct with branched microvasculature, Hepatoma derived cell line HepaRG was encapsulated in GelMA. CellTracker™ Red CMTPX Dye was used to stain HepaRG for visualization. After degradation, cell adhesion and removal of non‐adherent cells, a microvascularized liver tissue construct is formed and characterized by confocal epifluorescence microscopy using an image‐mosaic technique (Figure 5d). The mesoscopic construct can also be directly imaged using a Mesolens in confocal laser scanning mode (Figure S9), which is a novel approach for large‐scale imaging. Fabrication of microvascularized tissue construct provides a potential platform for pharmacological and toxicology testing, as well as proof of concept for fabricating complicated vascularized organs with multiple cell types.

## Conclusion

In the field of tissue engineering, a major roadblock is the fabrication of complex hierarchical structures to reconstitute the native multiscale vascular networks from arteries to blood capillaries.^[46]^ Constructing microvasculature is crucial for the formation of viable tissues or organs with complex hierarchical vascular networks. Currently, the major fabrication techniques for microvasculature could be divided into direct‐write technique and sacrificial technique.^[47]^ Direct‐write technique offers several advantages including low cost, ease of operation, time efficiency and its compatibility with a wide range of materials such as hydrogels and cell media. However, this technique is limited by its relatively weak mechanical properties, lower printing resolution, and the risk to cell damage due to shear stresses during the fabrication process. In contrast, sacrificial technique offers significant advantages, particularly in achieving high resolution, superior mechanical properties and enhanced structural fidelity, while maintaining high cell viability. Nevertheless, the technique imposes stringent requirements on the selection of sacrificial materials. Using existing sacrificial technique, it is still challenging to fabricate highly complex microvasculatures because the perfusion force during the required cell seeding step may damage the microchannels and result in larger and non‐uniform structures.

In this study, we have developed a PRINting Cell Embedded Sacrificial Strategy (PRINCESS) which offers the opportunity to tightly control the printing of complex microvascular networks. This is the first demonstration of direct cell printing to create microvasculatures, which eliminates the need for a subsequent cell seeding process and the associated deficiencies. We demonstrate the ability to print smaller than 100 μm microvasculatures, and we believe this new strategy can be further used to print smaller blood capillaries and achieve the replication of complex hierarchical tissue structures. Furthermore, the development of the PRINCESS could allow its use in fabricating thicker tissues with endothelialized microvasculature that would otherwise become necrotic.

DNA hydrogel has been used as a novel sacrificial biolubricant in our PRINCESS system, opening up new applications for supramolecular hydrogels. This is the first time to introduce the “lubrication” concept in the field of 3D bioprinting, which is essential to ensure the high viability of printed cells. Utilizing the controllable degradability of DNA hydrogel, we can fine‐tune its degradation time from minutes to weeks, making it suitable for both tissue engineering scaffolds and as a sacrificial material. Moreover, DNA hydrogel used here is a purely synthetic biomaterial. In comparison with other types of naturally occurring biomaterials such as collagen, gelatin and fibrin, synthetic DNA hydrogel has clear advantages with well‐defined chemical compositions, structures and porosities, while the mechanical, chemical, biological and degradation properties can be further designed and optimized. In addition, DNA hydrogel exhibits great molecular permeability, shear‐thinning, self‐healing and biocompatible properties, making it ideal for use in 3D cell culture,^[48]^ vaccine research,^[49]^ tissue engineering^[26]^ and 3D bioprinting.^[33]^ The shear‐thinning property of DNA hydrogel means that the hydrogel can be printed to form blood capillaries. Utilizing the shear‐thinning property, we have demonstrated a printed 70‐μm endothelialized microvasculature, breaking the limit of 100 μm. The self‐healing property of the supramolecular DNA hydrogel allows the fabrication of complex branched structures. In the past, although DNA materials have been demonstrated as tissue engineering scaffolds, the prohibitive cost of DNA prevents its wide applications. In our system, the application of the synthetic DNA hydrogel for sacrificial material requires extremely low volume of the DNA materials (e.g. 300 mg of DNA costing<$1,000 (USD) is required for printing a 1‐kilometer long microvasculature with 100 μm in diameter), paving the way for DNA materials to be used in practical tissue engineering applications.

GelMA hydrogel has been demonstrated to be compatible with many types of cells and support a wide range of tissue formation from vascular, cardiac, muscle, cartilage to bones.^[50]^ It is envisaged that our high‐resolution bioprinting technology could be extended to engineer other types of tissues with complex hierarchical networks. By printing endothelialized microchannels in engineered liver construct or kidney glomeruli, it could be used as dialysis systems to purify the blood by adsorbing and filtering accumulated toxins. By embedding different retina cells (such as pigment epithelial cells, visual cells and bipolar cells) in hydrogels and printing them layer‐by‐layer, we can potentially repair or fabricate these delicate retina structures. By printing neurocyte embedded hydrogels using our technique, we may print intricate neural networks for physiologically‐relevant in vitro models that are not currently possible. By encapsulating osteoblasts within the supporting bioinks, we can also obtain the microvascularized bone tissues, facilitating osteogenic differentiation and enhancing bone repair.^[51]^

Although the feasibility of PRINCESS technology has been proved in this work, there are still much work and many potential applications to be explored in the future studies. We envisage the PRINCESS can be investigated to bioprint 3D microvasculature in the combination with other biofabrication techniques such as FRESH (Freeform Reversible Embedding of Suspended Hydrogels) printing,^[52]^ SWIFT (sacrificial writing into functional tissue),^[53]^ and RIFLE (Rotational Internal Flow Layer Engineering)^[54]^ strategies. In addition, the functions of microvasculature such as blood delivery, substance exchange and endothelial barrier^[6]^ need to be verified by introduction of perivascular somatic cells and elongation of perfusion time. This would allow the creation of more realistic in vitro disease models and drug testing platforms, which may eventually eliminate the need for animal testing.^[55]^ In conclusion, PRINCESS is an innovative and effective strategy for the biofabrication of designable microvasculatures which overcomes the obstacles of current technologies and shows great potential in the field of tissue engineering, drug testing and biomedicine.

## Conflict of Interests

The authors declare no conflict of interest.

1

## Supporting information

As a service to our authors and readers, this journal provides supporting information supplied by the authors. Such materials are peer reviewed and may be re‐organized for online delivery, but are not copy‐edited or typeset. Technical support issues arising from supporting information (other than missing files) should be addressed to the authors.

Supporting Information

## Data Availability

The data that support the findings of this study are available in the supplementary material of this article.
