# Supplementary material for: Printing Cell Embedded Sacrificial Strategy for Microvasculature using Degradable DNA Biolubricant
Source: Angew Chem Int Ed Engl. 2024 Nov 27;64(12):e202417510. doi: 10.1002/anie.202417510 (PMC11914955; doi:10.1002/anie.202417510)
Supplement: Supplementary file 1 — Supporting Information [file ANIE-64-e202417510-s001.pdf]

Angewandte Chemie
Eine Zeitschrift der Gesellschaft Deutscher Chemiker

Supporting Information

Printing Cell Embedded Sacrificial Strategy for Microvasculature using Degradable DNA Biolubricant

J. Shi, Y. Wan, H. Jia, G. Skeldon, D. Jan Cornelissen, K. Wesencraft, J. Wu, G. McConnell, Q. Chen, D. Liu, W. Shu**

Printing Cell Embedded Sacrificial Strategy for Microvasculature using Degradable DNA Biolubricant

Jiezhong Shi# [a, b, c], Yifei Wan# [b], Haoyang Jia [a], Gregor Skeldon [b], Dirk Jan Cornelissen [b], Katrina Wesencraft [d], Junxi Wu [b], Gail McConnell [d], Quan Chen [e], Dongsheng Liu* [a], Wenmiao Shu* [b]

[a] Dr. J. Shi, Dr. H. Jia, Prof. D. Liu
Key Laboratory of Bioorganic Phosphorus Chemistry & Chemical Biology (Ministry of Education), Department of Chemistry
Tsinghua University
Beijing, 100084, China
E-mail: liudongsheng@tsinghua.edu.cn

[b] Dr. J. Shi, Y. Wan, Dr. G. Skeldon, Dr. D. J. Cornelissen, Dr. J. Wu, Prof. W. Shu
Department of Biomedical Engineering
University of Strathclyde
Glasgow G4 0NW, United Kingdom
E-mail: will.shu@strath.ac.uk

[c] Dr. J. Shi
SINOPEC Key Laboratory of Research and Application of Medical and Hygienic Materials
SINOPEC Beijing Research Institute of Chemical Industry Co., Ltd.
Beijing, 100013, China

[d] Dr. K. Wesencraft, Prof. G. McConnell
Department of Physics, SUPA
University of Strathclyde
Glasgow G4 0NG, United Kingdom

[e] Prof. Q. Chen
State Key Laboratory of Polymer Physics and Chemistry
Changchun Institute of Applied Chemistry, Chinese Academy of Sciences
Changchun 130022, China

#J. S. and Y.W. contributed equally to this work.

WILEY-VCH

Materials and Methods

Preparation of single strand DNA

All single strand DNA (see Table S1) was synthesized using a solid-phase method based on phosphoramidite chemistry[1]. First, a CPG loaded DNA synthesis column was put in Mermade-12 DNA synthesizer (Bioautomation company). Then the CPG ligated DNA oligomers were transferred into ammonia in 1.5 mL Eppendorf tubes. The mixture was heated to 60 °C for 3 h to separate DNA from CPG. After cooling down, the ammonia was removed in the rotation-vacuum-concentrator and the sample was purified by high performance liquid chromatography (HPLC, Agilent Technologies). Next, the sample was concentrated and the trifluoroacetic acid (CF3COOH) was added to remove the protective group DMT. Lastly, by removing salt in the ultrafiltration tubes, the purified DNA solution was obtained. The concentration of DNA solution was measured by Bio Cary-100 UV-Vis spectrometer (Varian). Required amount of DNA solution was lyophilized and stored at -20 ºC.

Polyacrylamide gel electrophoresis (PAGE)

10% ammonium persulfate (APS) and methylethylenediamine (TEMED) was introduced in 50 mL solution (Acrylamide: N,N'-Methylenebisacrylamide = 19: 1, 1×TBE buffer) to form a 10% native gel. DNA single strands and assemblies were mixed with 50% sucrose solution and loaded in the gel for electrophoresis. Electrophoresis was carried out at 4 ºC. After that, the gel was stained by “Stains All” for 10 min and then imaged with the ChemiDOC XRS + imager (Bio Rad).

Preparation of L1-Cy3

Cy3 CPG loaded DNA synthesis column was put in Mermade-12 DNA synthesizer (Bioautomation company) and synthesized using the solid-phase method based on phosphoramidite chemistry. All synthesis and purification methods are the same as normal single strand DNA. The sample was finally lyophilized and stored at -20 ºC and characterized by MALDI-TOF.

Preparation of DNA hydrogel

Stoichiometric amounts of DNA strands of the Y-scaffold (Y1, Y2, Y3, see Table S1) were lyophilized and dissolved in 1×PBS buffer (pH 7.4) to obtain a final concentration of 1 mM for each DNA strand. Stoichiometric amounts of the linker (L1, L2, see Table S1) were lyophilized and dissolved in 1×PBS buffer (pH 7.4) to give a final concentration of 1.5 mM for each DNA strand. Y-scaffold and linker solution were stabilized at 4℃ overnight prior to use. Then stoichiometric Y-scaffold and linker solution was mixed together to form the hydrogel.

WILEY-VCH

Characterization of the melting points of DNA assemblies

Y, L, Y1+L1, Y1+L1+Yc+Lc (Yc and Lc are complementary to the rest of Y1 and L1 except the sticky ends) were diluted to 5 μmol/L, and the melting point was characterized by Bio Cary-100 UV-Vis spectrometer (Varian). The absorbance was measured at wavelength of 260 nm with the temperature changing from 4 ºC to 95 ºC. The heating rate was 1 ºC/min. By simulating the data, we can get the melting point of the DNA assemblies.

Rheological characterization of DNA hydrogel

The mechanical properties of DNA hydrogel were tested by Kinexus rheometer (Malvern company). 40 μL of DNA hydrogel was put on the test bed. The diameter of the parallel fixture was 8 mm and the distance between test bed and parallel fixture was set to 0.15 mm. Parameters of the different model are followed:

Time scan test: 1% strain, 1 Hz, 25 °C, 3 min.

Strain scan test: 0.1%−1000% strain, 1 Hz, 25 °C.

Frequency scan test: 0.01-10 Hz, 1% strain, 25 °C.

Temperature scan test: 1% strain, 1 Hz, 4−65 °C.

Enzyme degradation of DNA hydrogel

Y scaffold was dissolved in 5 μL 1×PBS buffer (pH 7.4) with different concentration of Exo III (from 3 U/μL to 30 U/μL) to get a final concentration of 1mM. Linker was dissolved in 5 μL 1×PBS buffer (pH 7.4) to get a final concentration of 1.5 mM. Then two solutions were mixed together in the Eppendorf tube and put in 37 ℃ to explore the degradation time.

Lubrication property of DNA hydrogel

The instrument used for is the strain-controlled rheometer ARES-G2 at room temperature and the fixture is an 8 mm tapered plate. The test modes were dynamic and steady state. In this case, the dynamic mode applies a strain that varies sinusoidally with time, $\gamma(t)=\gamma_0\sin(\omega t)$, at which point the strain rate is $\dot{\gamma}(t)=\omega\gamma_0\cos(\omega t)=\dot{\gamma}_0\cos(\omega t)$, where the peak strain rate is $\dot{\gamma}_0=\omega\gamma_0$. The steady mode is when shear is initiated, which the shear rate $\dot{\gamma}$ is constant.

Calculation of shear stress for capillary printing

Paxton N. derived the following Power Law equation to characterise the shear thinning property.

$$\eta = K\dot{\gamma}^{n-1}$$

Where η denotes dynamic viscosity, γ denotes the shear strain rate. K and n are the constants representing shear thinning coefficient.

Besides, the shear stress reached high by using 40-80 μm glass capillaries to extrude DNA hydrogel. Kim H. fabricated a corneal structure with controllable aligned collagen fibrils by adjusting the needle size. To observe the collagen fibril alignment, he calculated that the shear stress concerning the radius of the nozzles:

$$\tau_{\text{wall}} = -K\left[\frac{Q_{\text{syringe}}}{R^3}\left(\frac{3n+1}{n}\right)\right]^n$$

τ_{wall} denotes wall shear stress, Q denotes volume flow rate, R denotes Needle radius. He proved that for a fixed volume flow rate, the shear stress is linearly proportional to the radius of the nozzle. In our study, the diameter of the glass capillaries is around 10-100 μm. The shear stress reaches over 1000Pa.

Synthesis of GelMA

GelMA was synthesized following a modified procedure previously described[2]. First, 10g gelatin (Type A, 300 bloom from porcine skin, Sigma) was dissolved in 100 mL Dulbelco's phosphate buffered saline (DPBS) at 60 °C for 2 h to obtain a final concentration of 10w/v% gelatin solution. The solution was kept at 37 °C to remove air bubbles. Then 1.25 mL of methacrylic anhydride (Sigma) was added drop-wise (10 min/mL) to the gelatin solution. The mixture was reacted at 55 °C for 3 h under stirring (1000 rpm/min). After which 100 mL DPBS was added to quench the reaction. To remove excess methacrylic acid and salts from the DPBS, GelMA solution was transferred to a 14 kDa molecular weight cutoff (MWCO) dialysis tubing (Sigma) and dialyzed in deionized (DI) water for 7 days (the water was changed twice daily). Finally, the GelMA was lyophilized and stored at −20 °C.

Characterization of the degree of methacrylation (DM) of GelMA

The methacrylation of gelatin was determined by ^{1}H-NMR spectroscopy. 10 mg GelMA powder was dissolved in 650 μL deuterium oxide and characterized using Bruker AVIII-HD-500 NMR Spectrometer. In the ^{1}H-NMR spectrum, the phenylalanine signal (6.50–8.00 ppm) represents the concentration of gelatin. If we normalized the phenylalanine signal, the degree of methacrylation was calculated as follows:

WILEY-VCH

$$DM(\%) = \left(1 - \frac{A[\text{lysine methylene in GelMA}]}{A[lysine\ methylene\ in\ gelatin]}\right) \times 100$$

In this equation, A[lysine methylene in GelMA] is the area of lysine methylene signals (3.10–3.25 ppm) in GelMA, and A[lysine methylene in gelatin] is the area of lysine methylene signals (3.10–3.25 ppm) in gelatin.

Preparation of DNA hydrogel sacrificial biolubricant

Y-scaffold and linker were dissolved in 15 μL 1×PBS buffer (pH 7.4) separately to obtain a final concentration of 1 mM Y-scaffold and 1.5 mM linker. The solutions were stabilized at 4 °C overnight prior to use. For Cy3-labeled DNA hydrogel, Stoichiometric amounts of Cy3-linker (L1-Cy3, L2) were dissolved in 1×PBS buffer (pH 7.4) to give a final concentration of 1.5 mM. 1 μL Cy3-linker was mixed with 14 μL normal linker and then stabilized at 4℃ overnight prior to use. For Exo III-embedded DNA hydrogel, 5 U/μL Exo III was introduced to Y-scaffold solution. For cell-embedded DNA biolubricant, 1×PBS buffer was changed to EGM-2 media, and HUVECs were removed from culture flasks and dispersed in the linker solution with the cell density of 4×10^6 cells/mL.

Preparation of GelMA supporting gel

Pure GelMA gel: 15 wt/v% GelMA powder was first dissolved in 1×PBS buffer or EGM-2 cell-culture media containing 0.3 wt% photoinitiator Irgacure 2959 (Sigma). The solution was sonicated at 37 °C until fully dissolved. Then the solution was kept at 37 °C overnight to remove air bubbles prior to use. *Cell-laden GelMA gel:* 15 wt/v% GelMA powder was dissolved in 1:1 EGM-2:William’s E cell-culture media containing 0.3 wt% Irgacure 2959 and the following steps are the same as pure GelMA bio-inks. HepaRG was removed from culture flasks and dispersed in the GelMA solution with a cell density of 2×10^6 cells/mL. The cell-laden GelMA bio-ink was pipetted up and down to mix thoroughly at 37 °C prior to use.

Cell culture and maintenance

Primary human umbilical vein endothelial cells (HUVECs) were purchased from Lonza and maintained in EGM-2 media (complete EGMTM-2 BulletKitTM, Lonza). Hepatoma derived cell line HepaRG was purchased from Thermo Fisher (HPRGC10) and maintained in Williams E medium (Gibco) supplemented with 10% FBS, 1% Penicillin/Streptomycin, 1% Glutamax (Gibco), 5 µg/ml insulin, (human recombinant, zinc solution, Gibco), and $5*10^{-5}$ M hydrocortisone hemisuccinate (Sigma

WILEY-VCH

Aldrich). Cells were kept in an incubator at 37 °C, in a humidified environment with 5% CO_2. Both cell cultures protocols followed the vendor's instructions.

Coverslips modification

The coverslips we used for microvasculature printing was pre-modified to generate chemical crosslinking with GelMA bio-ink. The coverslips were firstly sonicated in isopropyl alcohol, ethanol, and deionized separately for 5 min. Then the coverslips were dried and soaked in a 5% 3-(trimethoxysilyl)propyl methacrylate (Sigma) in toluene solution. The reaction was processed at 60 °C overnight. Finally, the coverslips were rinsed with isopropyl alcohol and dried prior to use.

Preparation of glass capillary with different diameter

Glass capillary (OD = 1 mm) was pulled using P-1000 Micropipette pullers (Sutter Instrument). The parameters were set as follows:
Ramp = 488, Heat = 518, Pull = 0, Vel = 150, Time = 0, Pressure = 500
After pulling, the glass capillary with long tapered tip was obtained. The tip was cut at different position under microscope to obtain different diameters.

3D bioprinting

All the structures were printed using a custom-built, three-axis (X-Y-Z), bioextrusion-based 3D cell printer. Briefly, the printer runs by coordinating the motion of a mechanically-driven syringe. Biolubricant was loaded in the syringe and printed on a stationary Z-platform, which moves downwards allowing the structures to be printed layer-by-layer from the bottom up. Before printing, all equipment was sterilized by wiping down with 70% ethanol, and sterility was maintained during printing process by placing the bioprinter in a laminar flow cabinet.
Engineered tissue constructs were produced by printing sacrificial DNA hydrogel and cast with GelMA. Initially, 15 μL Exo III-laden Y-scaffold and 15 μL HUVECs-laden linker were loaded in a 100 μL glass syringe (Hamilton company) alternating every 5 μL and mixed thoroughly to form the DNA hydrogel biolubricant. The glass capillary with varying diameters was attached to the glass syringe through a 1mm compression fitting (55750-01, Hamilton company). Then the syringe was loaded into the bioprinter and the 3D structures were printed onto a coverslip. After printing, the HepaRG-laden GelMA supporting gel was deposited on the printed vascular structure and the entire structure was exposed to UV light (UV LED curing system, 365 nm, 60 s) for GelMA crosslinking.

Endothelialization

WILEY-VCH

The structure was put in the incubator for DNA hydrogel degradation and HUVECs attachment. After 24 hours, the structure was turned upside down and incubated at 37 °C for another day to allow HUVECs to attach to all sides of the channel. Then, non-adherent cells were flushed out of the channels with EGM-2 media and the tissue constructs with branched microvascularization were obtained.

Cell viability assay

Cell viability of HUVECs cultured in a 24-well plate with 150U Exo III after 3 d was determined by fluorescent live/dead assay. Initially, 1mL 1×PBS was added to the well. Then, 2μL Propidium Iodide (Sigma Aldrich) stock solution (2mg/mL) was added to the PBS solution. After incubating at room temperature for 30 min, 1μL Fluorescein Diacetate (Sigma Aldrich, 5mg/ml, dissolved in acetone) was added, and the sample was imaged using the inverted fluorescence microscope (Nikon Eclipse TE300).

Microscopic Imaging

For imaging, HUVECs were labeled with CellTracker™ Green CMFDA Dye and HepaRG was labeled with CellTracker™ Red CMTPX Dye (Invitrogen) prior to use. Printed structure and microvascularized branched tissue constructs were imaged using the inverted fluorescence microscope (Nikon Eclipse TE300) or confocal (Leica SP5) epifluorescence microscopes.

Mesoscopic Imaging

For mesoscopic imaging, specimens were prepared in the same way as for microscopic imaging. The specimens were mounted in a large custom-designed imaging chamber and were mounted in PBS for imaging. Laser scanned confocal mesoscopic imaging of all specimens was performed on a Mesolens laser scanning confocal instrument. Details of the Mesolens are reported elsewhere[3] so we describe the setup here only briefly. The Mesolens is a custom-designed objective lens that has the unique combination of low magnification (4x) and high numerical aperture (0.47). It is chromatically corrected across the entire visible spectrum and is compatible with multiple immersion fluids. It has a total maximum volume of capture of over 100 cubic millimetres (6 mm x 6 mm x 3 mm), and the high numerical aperture gives sub-cellular resolution images of the full volume without stitching or tiling. In this work, images were acquired with simultaneous laser excitation from two excitation wavelengths. A wavelength of 488 nm at an average power of 25 mW from the oscillator was used for excitation of fluorescence from CellTracker™ Green CMFDA Dye, and a wavelength of 561 nm with an average power of 15 mW from the oscillator was served to excite fluorescence from CellTracker™ Red CMTPX Dye. Laser scanning was achieved using a custom galvo mirror arrangement, and the lasers were focused into the specimen using the Mesolens. Fluorescence was collected with the Mesolens in a

WILEY-VCH

descanning epifluorescence configuration and was separated into two separate wavelength bands using a 550 nm dichroic filter. Fluorescence emission longer than 550 nm, corresponding to the signal from CMTPX Dye, was passed through a 600 nm long-pass filter to reject any stray 561 nm laser light and was detected using one photomultiplier. Fluorescence emission at wavelengths shorter than 550 nm (i.e. from CMFDA Dye) was passed through a 525/39 nm filter to reject any stray 488 nm laser light, and was detected using a second photomultiplier. Image acquisition was performed using custom 'Mesoscan' software. Images were obtained across the entire 6 mm x 6 mm field of view with Nyquist sampling (3 pixels/μm) and a pixel dwell time of 0.5 μs, and a frame average of n=2 was applied to reduce noise. A z-stack was obtained by moving the specimen stage of the imaging system in 3 μm increments between successive images. A total of 34 images was obtained for each channel, imaging 102 μm deep into the specimen.

Cost calculation for printing a 1-kilometer long microvasculature with 100 μm in diameter

(1) The volume of DNA hydrogel (V) used for 1-kilometer long microvasculature with 100 μm in diameter is:

$$V = \pi R^2 \times \mathrm{L} = 3.14 \times (5 \times 10^{-3})^2 \times 10^5 \text{ mL} = 7.85 \text{ mL}$$

(2) Because the solid content of the DNA hydrogel used is 3.8 w/w%, the mass of DNA (m) used in the above volume of DNA hydrogel is:

$$m = 3.8\% \times 1\frac{g}{mL} \times 7.85mL = 0.3 \text{ g} = 300 \text{ mg}$$

(3) The length of DNA used is 40 bases with molecular weight (M_w) of around 12000 g/mol, so the amount (n) of DNA used is:

$$n = \frac{m}{M_W} = \frac{0.3g}{12000\frac{g}{mol}} = 2.5 \times 10^{-5}\, mol = 25\, \mu mol$$

(4) For the synthesis of 25 μmol DNA, the feeding amount is about 250 μmol, which will consume CPG of 10 g and the monomer of 50 g. The cost of raw materials was estimated to be \$ 400 (USD) by our supplier DNA CHEM company. Adding the cost of purification, equipment, labor and electricity, the total cost could be controlled within \$1,000 (USD).

Supplementary Figures and Tables

Table S1. ssDNA sequences of DNA hydrogel (Red colored sequences are sticky ends).

Y1	5'-CGATTGACTCTCCACGCTGTCCTAACCATGACCGTCGAAG-3'
Y2	5'-CGATTGACTCTCCTTCGACGGTCATGTACTAGATCAGAGG-3'
Y3	5'-CGATTGACTCTCCCTCTGATCTAGTAGTTAGGACAGCGTG-3'
L1	5'-GAGAGTCAATCGTCTATTCGCATGAGAATTCCATTCACCGTAAG-3'
L2	5'-GAGAGTCAATCGCTTACGGTGAATGGAATTCTCATGCGAATAGA-3'

WILEY-VCH

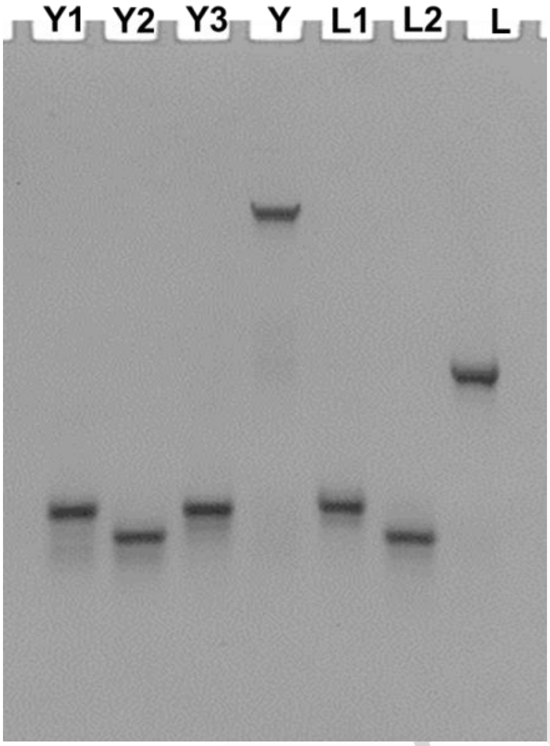

Figure S1. 10% Native PAGE (19:1) analysis of ssDNA, Y-scaffold and linker in 1×PBS buffer.

WILEY-VCH

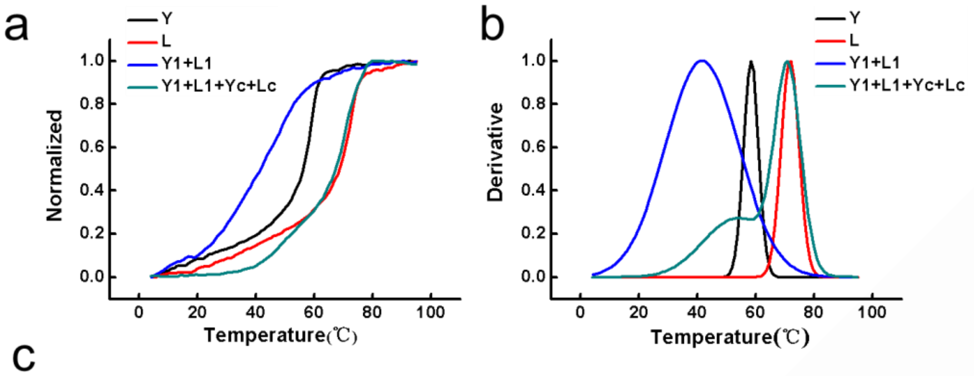

c

Sample	Y	L	Y1+L1	Y1+L1+Yc+Lc
Melting point (℃)	58.3	72.1	41.7	53.0

Figure S2. UV-Vis spectra of DNA assemblies. a. Relationship of UV-Vis spectra of Y, L, Y1+L1, Y1+L1+Yc+Lc with temperature; b. Derivation of UV-Vis spectra for the melting points of Y, L, Y1+L1, Y1+L1+Yc+Lc (Yc and Lc are complementary to the rest of Y1 and L1 except the sticky ends); c) Melting points of Y, L, Y1+L1, Y1+L1+Yc+Lc.

WILEY-VCH

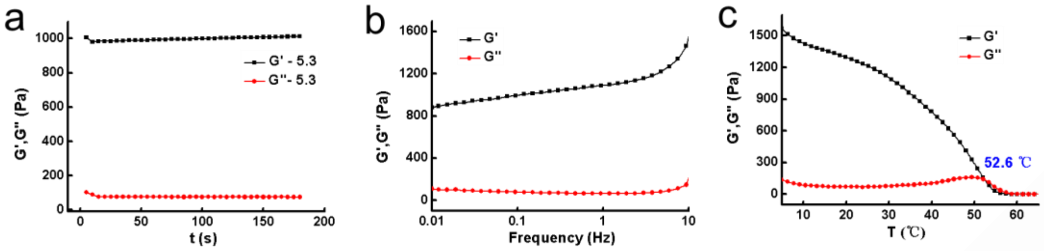

Figure S3. Rheological characterization of 3.8 wt% DNA hydrogel in 1×PBS buffer: a. time-scan test, b. frequency-sweep test, c. temperature-ramp test.

WILEY-VCH

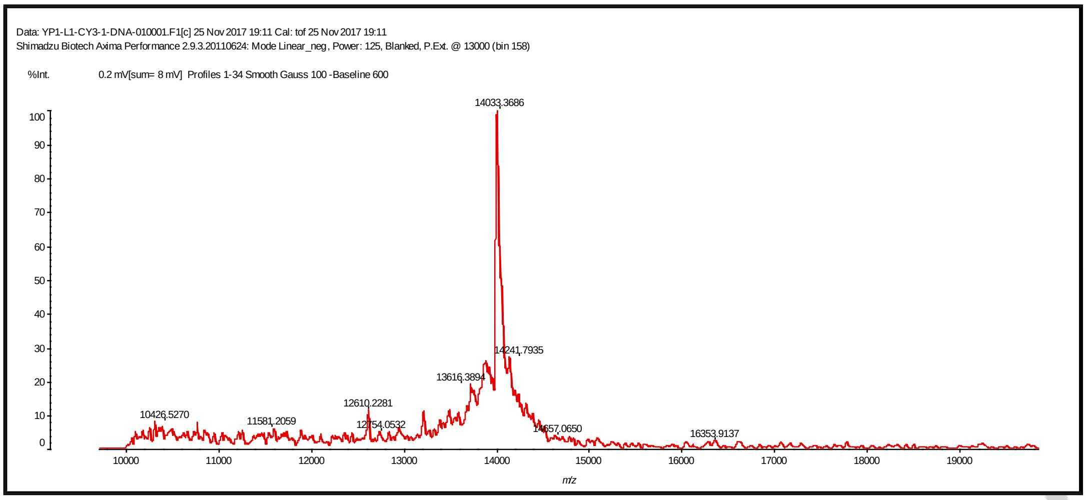

Figure S4. MALDI-TOF characterization of L1-Cy3 and the molecular weight was found to be 14033.

WILEY-VCH

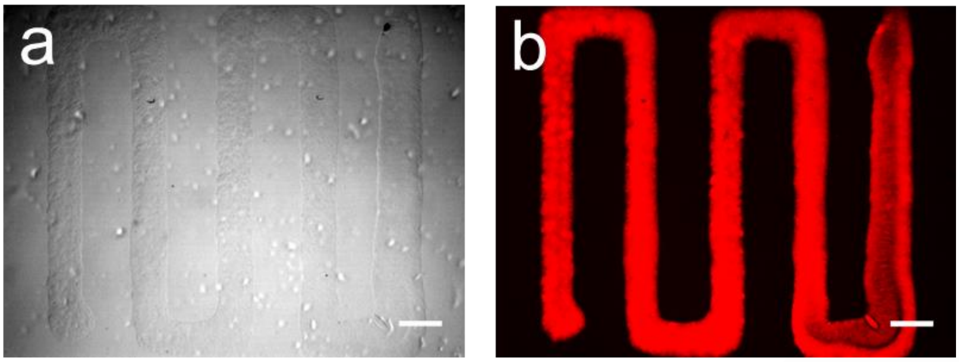

Figure S5. Optical (a) and Fluorescent (b) images of printed Cy3 labeled DNA hydrogel cast by 15 wt% GelMA in the incubator after 7 d. Scale bar 200 μm

WILEY-VCH

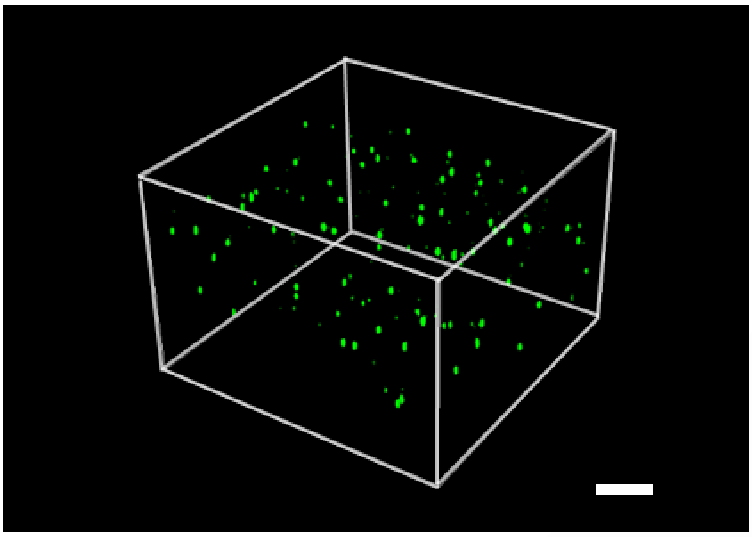

Figure S6. Live/dead staining image of HUVECs printed in the DNA hydrogel. Scale bar 200 μm;

WILEY-VCH

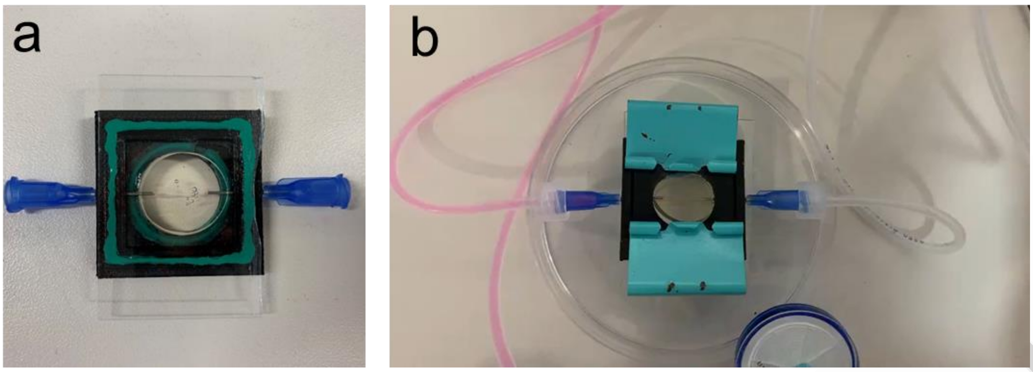

Figure S7. Pictures of perfusion equipment and system.

WILEY-VCH

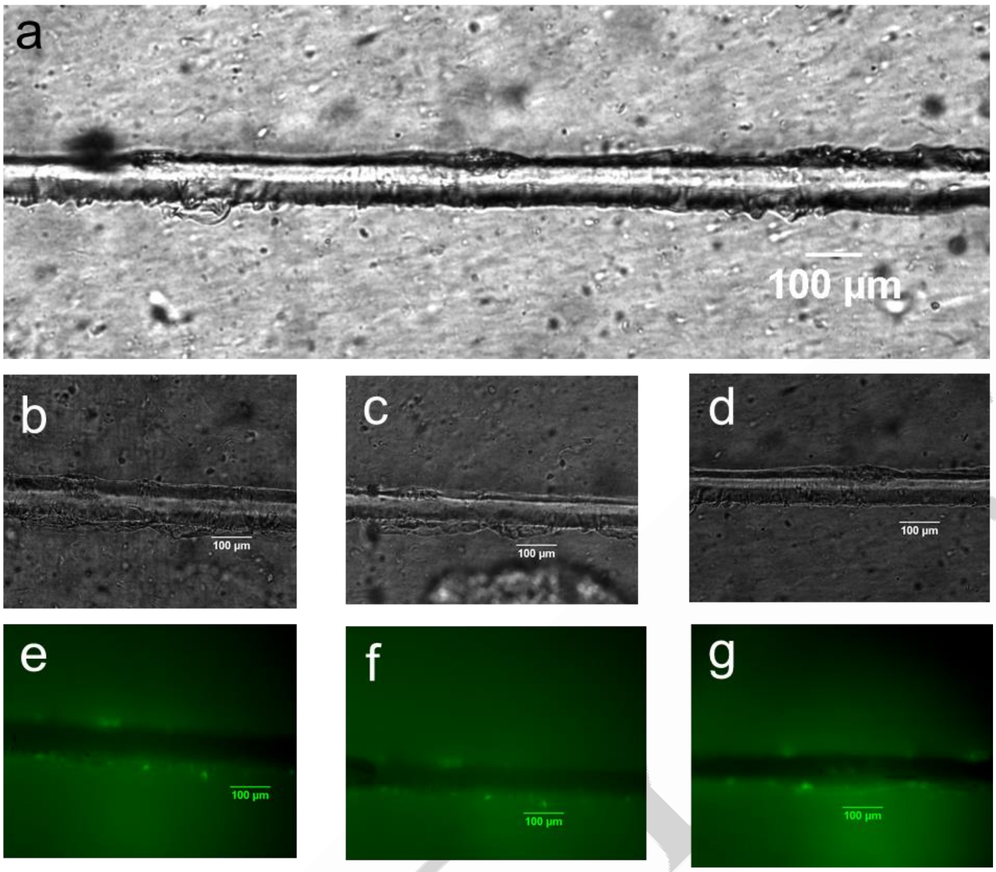

Figure S8. Images of microvasculature after perfusion. a. Optical image of microvasculature before perfusion; b-d. Optical images of HUCEVs after perfusion for 1, 5 and 7 days; e-g. Fluorescent images of HUCEVs after perfusion for 1, 5 and 7 days.

WILEY-VCH

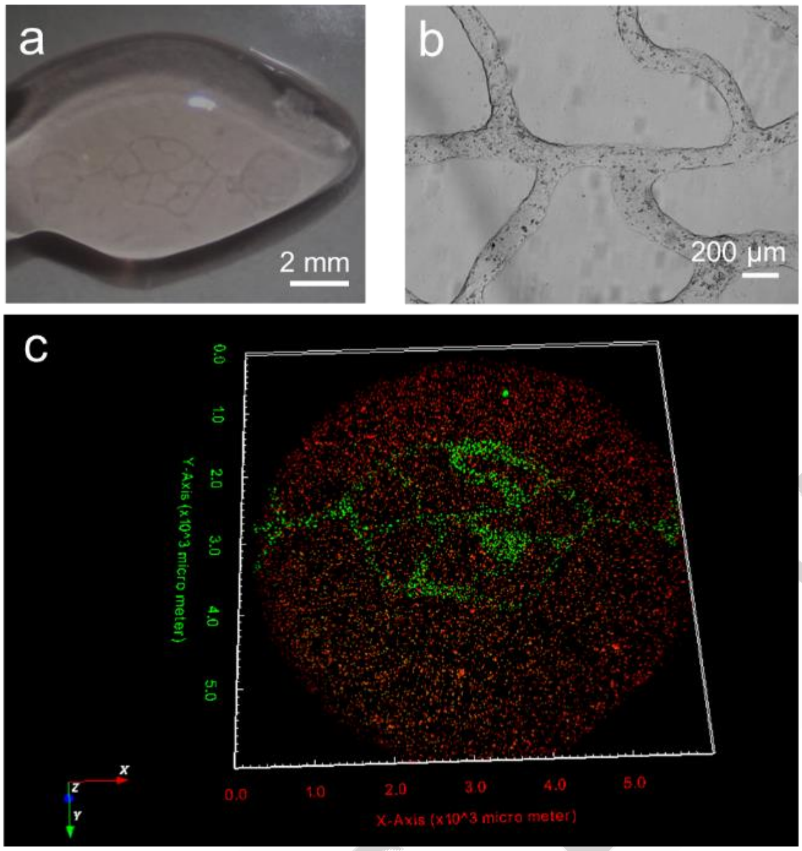

Figure S9. Images of microvascularized construct. a. Picture of microvascularized construct; b. Optical image of microvascularized construct; c. Mesoscopic fluorescence image of microvascularized liver tissue construct.

WILEY-VCH

Supplementary References

[1] S. J. Horvath, J. R. Firca, T. Hunkapiller, M. W. Hunkapiller, L. Hood, *Methods enzymol.* **1987**, *154*, 314.

[2] A. I. Van Den Bulcke, B. Bogdanov, N. De Rooze, E. H. Schacht, M. Cornelissen, H. Berghmans, *Biomacromolecules* **2000**, *1*, 31.

[3] G. McConnell, J. Trägårdh, R. Amor, J. Dempster, E. Reid, W. B. Amos, *eLife* **2016**, *5*, 18659.

WILEY-VCH
